# Polyamine–Drug Conjugates: Do They Boost Drug Activity?

**DOI:** 10.3390/molecules28114518

**Published:** 2023-06-02

**Authors:** Filippo Basagni, Giambattista Marotta, Michela Rosini, Anna Minarini

**Affiliations:** Department of Pharmacy and Biotechnology, Alma Mater Studiorum—University of Bologna, Via Belmeloro 6, 40126 Bologna, Italy; filippo.basagni2@unibo.it (F.B.); giambattista.marott2@unibo.it (G.M.); michela.rosini@unibo.it (M.R.)

**Keywords:** polyamine, drug conjugate, antitumor, antimicrobial, neuroprotective

## Abstract

Over the past two decades, the strategy of conjugating polyamine tails with bioactive molecules such as anticancer and antimicrobial agents, as well as antioxidant and neuroprotective scaffolds, has been widely exploited to enhance their pharmacological profile. Polyamine transport is elevated in many pathological conditions, suggesting that the polyamine portion could improve cellular and subcellular uptake of the conjugate via the polyamine transporter system. In this review, we have presented a glimpse on the polyamine conjugate scenario, classified by therapeutic area, of the last decade with the aim of highlighting achievements and fostering future developments.

## 1. Introduction

**Figure 1:**
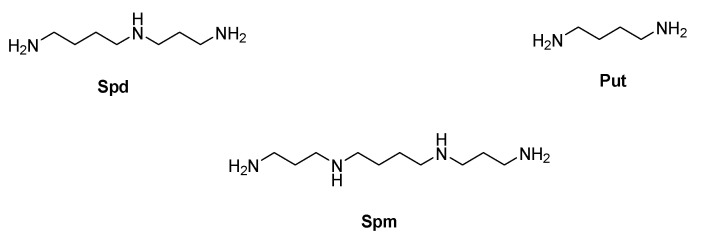
Chemical structures of spermine, spermidine and putrescine.

Polyamines are polycationic aliphatic molecules consisting of a hydrocarbon-made skeleton with at least two amino groups interposed where the number of nitrogen atoms, length and architecture account for the different biological activities. The three major polyamines triamine spermidine (Spd), tetramine spermine (Spm) and their precursor putrescine (Put, Figure 1) are almost ubiquitous and widely distributed in both prokaryotic and eukaryotic cells [1,2,3].

At physiological pH, the amino functional groups are positively charged and can interact with negatively charged macromolecules such as RNA, DNA, protein and phospholipid. Polyamine’s cellular content is tightly regulated given its involvement in several basic cellular functions such as growth, motility, apoptosis, differentiation and response to exogenous stress [4]. Due to the essential role played in cellular machinery, alterations of the polyamine pool classically represent a marker of cellular impairment. Some polyamines can be introduced through diet, but the three major natural polyamines (Put, Spd and Spm) are basically biosynthesized in the cytoplasm of all cells. The key brick of the polyamine pathway is Put, which mainly derives from decarboxylation of ornithine catalyzed by ornithine decarboxylase (ODC). From Put, two sequential steps of aminopropyl group addition, added by decarboxylated S-adenosylmethionine (dcSAM), yield Spd and Spm, respectively catalyzed by spermidine synthase and spermine synthase. On the contrary, catabolic processes involve oxidative steps that release reactive oxygen species (ROS) as by-products. Briefly, Spm is reconverted to Spd with spermine oxidase (SMOX) or through an acylation–oxidation mechanism mediated by spermidine/spermine N1-acetyl transferase 1 (SSAT) and polyamine oxidase (PAOX), respectively, which works also for Spd-to-Put reconversion. The scenario herein briefly reported is far better meticulously tuned thanks to the involvement of several other enzymes and cofactors, besides feedback mechanisms guided by the same substrate concentration [5]. Furthermore, another key component of the polyamine pathway is represented by the polyamine transport system (PTS) because physiologically protonated polyamines are not able to passively diffuse through cell membrane. The PTS mechanism is not yet fully elucidated but seems to work with a complex system of endocytosis or vesicle-mediated transport. PTS together with anabolic and catabolic enzymes constitute an optimized regulatory system for polyamine cellular concentration, while alterations of this balance are related to peculiar or pathological cellular conditions. Particularly, PTS upregulation is observed in physiological proliferating cells and further exacerbated in carcinogenic cells, while polyamine biosynthesis decreases with aging, making cells more susceptible to exogenous stimuli [6,7,8].

Intrinsic biological activities of natural polyamines paired to the essential role played by polyamines for cellular homeostasis in pathophysiological conditions paved the way for a plethora of drug discovery approaches targeting the polyamine pathway at multiple levels [9]. Particularly, conjugation of polyamines with bioactive molecules has been envisioned as a promising strategy to improve their potential therapeutic efficacy for several objectives: (1) targeting specific tissue to deliver bioactive payloads with increased selectivity thanks to peculiar up/downregulation of polyamines in diseased conditions; (2) exploiting PTS to increase cell entrance for compounds with suboptimal PK properties; (3) optimizing target engagement by leveraging its polycationic architecture; (4) simultaneous modulation of enzymes involved in polyamine metabolism and additional target(s), resulting in a multifaceted mechanism of action. The same approach can also be applied to identify new molecular tools that could help to clarify the role of polyamines in different diseased conditions [10,11]. In this review, we have presented a glimpse on the polyamine conjugate scenario, categorized by pathological area, of the last decade with the aim of pointing out achievements and fostering future developments. Due to polyhedral activities of polyamines, some conjugates were evaluated in different diseased conditions, but herein are reported only with reference to their best observed therapeutic efficacy, for clarity. Furthermore, it is important to clarify that the conjugated fragment, by definition, should bring additional biological activities to the polyamine portion, differing from simple polyamine analogues that are not covered here. For the same reason, all of the published derivatives where polyamine functional groups were inserted as simple linker or structural modification within structure–activity relationship studies, and not as intended for their biological activities, are not reported here because that is beyond the scope of the conjugate approach.

## 2. Antitumor Agents

The finding of increased polyamine concentration in cancer cells, as well as in other diseased tissues, paved the way for several drug discovery campaigns involving polyamine trafficking as a promising target. Particularly, upregulation of polyamines' biosynthetic enzymes and downregulation of their catabolic enzymes are strictly related to malignant cell proliferation and tumorigenesis [12]. Furthermore, there is experimental evidence that dysregulated polyamines trigger several oncogene pathways or improve the malignancy of tumors, to the extent that polyamines and their metabolites have been regarded as cancer biomarkers [13]. Polyamines are required for cell proliferation, and this fact makes cancer cells particularly sensitive to polyamine depletion. In addition, cancer cells strongly rely on exogenous polyamines, which are imported through an overactivated PTS, because they are unable to biosynthesize enough polyamines [14]. Based on these premises, the polyamine metabolic pathway has been envisioned as a promising target for antitumor therapeutic treatments. Particularly, several inhibitors of polyamine biosynthesis have been developed as well as modulators of the polyamine transport system, catabolism and polyamine cellular content for cancer chemotherapy or chemoprevention [5,15]. In addition to the polyamine analogue strategy, the overactivation of PTS in cancer cells was exploited to increase the antiproliferative activities of some anticancer agents through the development of polyamine conjugates with the aim of leveraging a polyamine driving force for tailored delivery [16]. In this context, the pioneering breakthrough of F14152, defined as “polyamine-vectorized anticancer drug” with its promising preclinical and clinical progression, paved the way for the polyamine conjugate approach in anticancer drug discovery [17,18,19]. Herein, we have dissected the realm of antitumor polyamine conjugates by categorizing them based on the nature of the conjugate portion, which mainly comprises natural (or nature-inspired) scaffolds or synthetic polycyclic cores such as naphthalimides.

### 2.1. Natural Scaffolds

**Figure 2:**
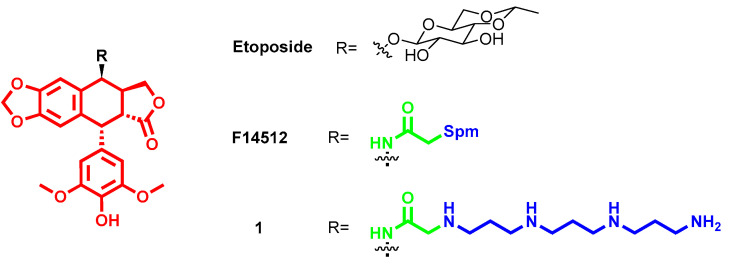
Etoposide and its polyamine-conjugated derivatives. The bioactive core is highlighted in red, the polyamine chain in blue, and the linker portion in green.

Nature has always represented a source of inspiration, but also production for drug development, and this applies also for anticancer drugs. The above-mentioned success of F14512 relies on the therapeutic properties of etoposide, which is an anticancer drug that acts by inducing topoisomerase II-mediated DNA cleavage, and it is used to treat a wide spectrum of human cancers. Particularly, in F14512, the podophyllotoxin core is linked to an Spm moiety at C4, instead of etoposide’s sugar moiety, as a cancer cell delivery vector and DNA anchor thanks to its positive charges (Figure 2). The polyamine conjugation notably increased both F14512 uptake in cancer cells and its cytotoxic activity with respect to etoposide, both in vitro (10 times more potent topoisomerase II poison thanks to an increased DNA affinity) and in vivo without showing any toxicity issues [16]. The presence of the polyamine chain in F14512 resulted in tighter binding and increased stability of the ternary topoisomerase II-drug-DNA complex [20]. This was further confirmed through the development of F14512’s analogues by substituting the Spm tail with other polyamines that vary in length and number of nitrogen atoms [21]. Particularly, the polyamine moiety boosts the drug binding and stabilization of enzyme–DNA complex, thus showing higher inhibitory properties toward topoisomerase II for all new derivatives (except for one bearing an inner ether chain) with respect to etoposide, albeit remaining less potent than F14512. Among the series, compound **1** (Figure 2) with a 3-3-3 polyamine chain, most similar to the F14512 spermine, emerged as the most potent with an IC_50_ = 35 µM vs. 30 µM for F14512, but still four times better than etoposide (IC_50_ = 120 µM) [21]. Further insights revealed that all polyamine hybrids demonstrated higher abilities to induce double-stranded (ds) DNA breaks with isoform topoisomerase IIβ than topoisomerase IIα. Generally, all conjugates triggered DNA cleavage to a higher extent than etoposide with topoisomerase IIα, and even more with topoisomerase IIβ. Computational simulations revealed that the presence of a glutamine residue in topoisomerase IIβ, instead of methionine in topoisomerase IIα, and the resulting interaction with polyamine tail are behind the difference in the enhanced specificity of these conjugates toward the former isoform [22].

**Figure 3:**
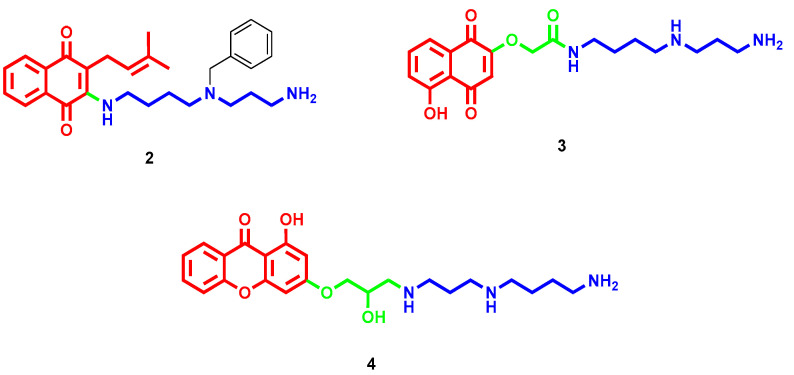
Naphthoquinone– and xanthone–polyamine conjugates. The bioactive core is highlighted in red, the polyamine chain in blue, and the linker portion in green.

1,4-Naphthoquinone constitutes another nature-derived pharmacophore endowed with cytotoxic properties; thus, several clinically used chemotherapeutics possess a quinone scaffold. By inserting a substituted Spd tail on the naphthoquinone core of lapachol, a remarkable increase in selective cytotoxicity for glioblastoma cells was observed while preserving astrocytes [23]. The IC_50_ value for polyamine-conjugate **2** (Figure 3) dropped to 6.6 µM for the GBM95 cell line and to 4.3 µM for the U87MG cell line (two models of in vitro glioblastoma) compared to 23.4 µM and 18.4 µM, respectively, with starting naphthoquinones. Furthermore, at 50 µM, **2** significantly reduced glioblastoma tumor invasion. Topoisomerase IIα inhibition notably increased for the reported polyamine conjugate, and this partially accounted for its cytotoxic profile in cancer cells [23]. Contrarily, the Spd derivative of 5-hydroxynaphthoquinone **3** (Figure 3) retained promising anticancer activity in the low µM range, but without any significant improvement over the starting unconjugated scaffold [24].

Driven by the promising anticancer properties of gambogic acid, a small series of xanthone-polyamine hybrids were developed with the aim of defining their mechanism of action at topoisomerase IIα [25]. The most potent compounds of the series, featured by an Spd or Spm side chain, act as catalytic inhibitors with IC_50_ values of around 1–3 µM, while the xanthone core and separated polyamine are completely inactive. Particularly, in-depth analyses on compound **4** (Figure 3) highlighted the ability to inhibit the strand passage step of topoisomerase activity by suppressing the ability of DNA to promote the rate of ATP hydrolysis. This was possible because of its interaction in the proximity of the DNA cleavage active site even though it has almost no effect on the cleavage reaction [25].

**Figure 4:**
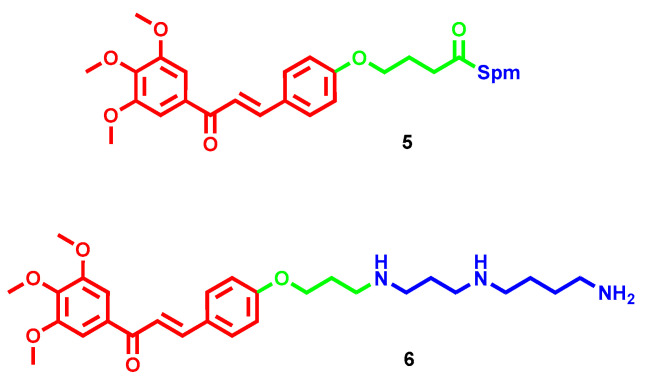
Chalcone–polyamine conjugates. The bioactive core is highlighted in red, the polyamine chain in blue, and the linker portion in green.

To efficiently deliver in cancer cells the well-known antiproliferative effect of chalcones, the chalcone scaffold was connected to different polyamines through an amido linker, and the cytotoxicity of the conjugates was evaluated in different colorectal and prostatic cancer cell lines [26]. Surprisingly, all of the polyamine conjugates exerted moderate antiproliferative effects in comparison to the parent chalcones. Among them, the Spm derivatives emerged as the most promising, with compound **5** (Figure 4) acting as one of the most active in prostatic cancer cell lines (IC_50_ = 34 µM in PC-3, and IC_50_ = 35.4 µM in DU-145) but also in the colorectal HCT-116 (IC_50_ = 33.8 µM) [26]. A follow-up series where the amido linkage was replaced with an amino linkage led to an overall increase in the antiproliferative activities, similar to those of parent chalcone [27]. In this case, the Spd derivative **6** (Figure 4) emerged as the most potent in all tested human cancer cell lines, with IC_50_ ranging from 8 to 13 µM. It showed a tissue-specific effect on cell cycle progression, with induced cell cycle arrest in the G1 phase for colorectal cells or in the G2 phase for prostate cells, combined with apoptosis induction [27].

**Figure 5:**
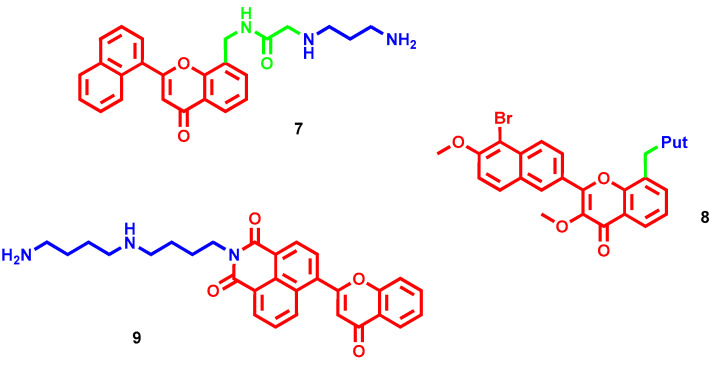
Flavonoid–polyamine conjugates. The bioactive core is highlighted in red, the polyamine chain in blue, and the linker portion in green.

Flavonoids constitute another class of natural compounds, chemically characterized by a chromone core, endowed with a plethora of beneficial properties, including an antiproliferative effect. In a small series of polyamine–flavonoid conjugates, compound **7** (Figure 5) exerted the best selective cytotoxic effect, albeit moderate, in liver tumor cells (IC_50_ = 65 µM for HepG2 and 32 µM for H22) compared to normal liver cells [28]. The potency mildly increased if aspirin was used as adjuvant (IC_50_ = 59 µM for HepG2 and 21 µM for H22). Furthermore, it also showed a dose-dependent antimetastatic effect by inhibiting tumor cell invasion and migration, and pro-apoptotic properties as confirmed by the enhanced level of caspases, ROS and other apoptotic-related factors. In vivo, when given alone, **7** at 20 mg/kg exhibited only a moderate tumor inhibition rate (around 40%), which notably increased when co-administered with aspirin 20 mg/kg (69%). The same anticarcinogenic effect was also confirmed in lung metastasis, with inhibition rates of 29.3% alone and 59.9% with aspirin [28]. Further chemical optimizations led to compound **8** (Figure 5) featuring a Put tail with enhanced antitumor activity both in vitro (IC_50_ = 7.10 µM for HepG2) and in vivo (69.1% inhibition of tumor progression at 40 mg/kg) [29]. The naphthalene-chromone core provided moderate fluorescence that allowed defining a non-specific subcellular localization for **8** (i.e., mainly mitochondria and endoplasmic reticulum), from which the complex cytotoxic profile originated. Compound **8** proved to induce apoptosis, mainly activating caspases 3, 8 and 9, but also activated autophagy processes that are usually adverse to apoptotic pathways [29].

Encouraged by the promising results obtained with flavonoids, the same authors reported a new series of chromone–polyamine conjugates where a naphthalimide core, endowed with anticancer and bioimaging properties, was inserted between the two scaffolds [30]. The hit compound **9** (Figure 5) bearing an homospermidine chain inhibited hepatoma proliferation in a dose-dependent manner, as well as preventing its migration, with a marked selectivity over a healthy hepatocyte cell line. In a tumor animal model, **9** at 3 mg/kg strongly suppressed tumor growth and metastasis better than amonafide, a naphthalimide-based antitumor agent, at 5 mg/kg (60.4% vs. 48.1% and 77.9% vs. 41.4%, respectively). Thanks to the fluorescent properties of the naphthalimide moiety, it was possible to visualize **9**’s cellular route and mechanism of action: it was taken up partially through PTS, without affecting cellular polyamine metabolism, and localized in the mitochondria where it triggered tumor-selective oxidative stress, inducing apoptosis and migration inhibition of hepatoma cells [30].

Triterpenes constitute a huge family of natural products derived from plants endowed with important biological properties. Among others, the intrinsic antiproliferative effect, paired with easy membrane permeation and a feature of different anchor points, guided the development of triterpene–polyamine conjugates for potential anticancer efficacy. The same strategy was followed in pursuing antimicrobial triterpene–polyamine conjugates, achieving more promising experimental outcomes. Therefore, herein, only major results for these derivatives are reported, and greater space will be devoted to triterpene conjugates in the antimicrobial section.

**Figure 6:**
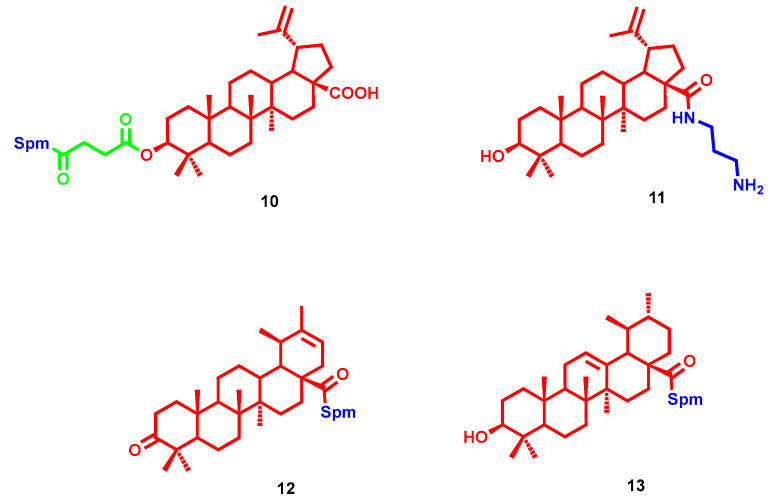
Triterpene–polyamine conjugates with promising anticancer profile. The bioactive core is highlighted in red, the polyamine chain in blue, and the linker portion in green.

Betulinic acid was identified as a suitable building block for anticancer polyamine conjugates, and different polyamines were attached at OH in C3 through a hemisuccinate linker and at the carboxy functional group in C28. Generally, amido-derivatives conjugated at C28 exerted higher cytotoxicity in cancer cell lines but were equipotent toward normal human fibroblast cell line, highlighting low selectivity. Regarding the C3-attached series, spermine-bearing **10** (Figure 6) displayed important cytotoxicity toward human T-lymphoblastic leukemia cells (IC_50_ = 5.2 ± 2.3 µM) with moderate selectivity (IC_50_ = 42.9 ± 3.8 µM toward normal human fibroblasts) [31]. Compound **10** was also selected to evaluate its antimicrobial activity (see Antimicrobial paragraph). More recently, another series of betulinic acid conjugates were reported with polyamines of different lengths attached at C28 over an amido functional group. In this case, the shorter tail of **11** (Figure 6) turned out as the preferred substitution for antiproliferative activity. In a panel of 60 human tumor cell lines (ranging from leukemia to ovarian cancer, melanoma, lung cancer, colon cancer, etc.), compound **5** showed GI_50_ values (concentration of the compound causing a 50% decrease in net cell growth) ranging from 1.09 µM to 13.20 µM [32]. Similarly, Spm attachment at C28 of heterobetulonic and ursolic acid (**12** and **13** respectively, Figure 6) produced important anticancer properties among a series of triterpenoid acid conjugates. Particularly, they exerted low micromolar cytotoxicity in a panel of cancer cell lines (IC_50_ = 2.8–4.8 µM for **12** and IC_50_ = 6.3–8.1 µM for **13**), confirming also comparable toxicity toward normal human fibroblast cell line, as emerged from previous betulinic acid derivatives [33]. Moreover, several ethylenediamide tails were inserted at C28 of oleanolic, betulinic or maslinic acid, achieving high cytotoxicity, albeit with low selectivity over non-cancer cells, by triggering apoptosis [34,35,36]. Furthermore, among oleanolic conjugates, by varying the length of the diaminoalkyl chain, cytotoxicity was only partially affected [36]. Lastly, stigmasterol conjugation yielded a suboptimal anticancer profile, where the most potent Spm derivative had a highlighted IC_50_ > 30 µM [37].

**Figure 7:**
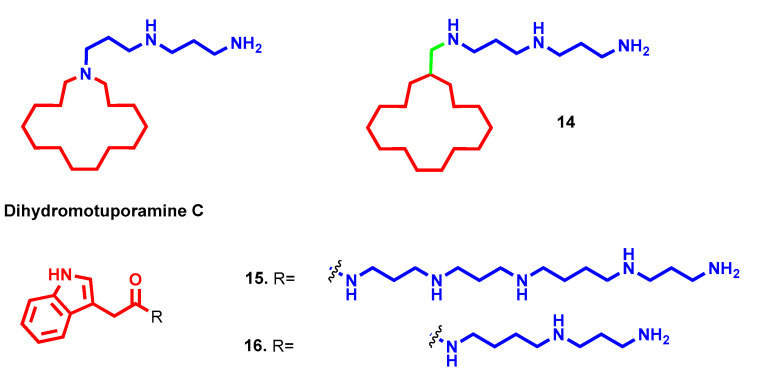
Motuporamine and polyamine toxin derivatives. The lipophilic head is highlighted in red, the polyamine chain in blue, and the linker portion in green.

Motuporamines are natural products isolated from a sea sponge and are endowed with remarkable antimetastatic efficacy and chemically characterized by a macrocycle with a polyamine tail appended. Particularly, dihydromotuporamine C (Figure 7), with a norspermidine embedded into the cycle, is powered with antimigration and antiangiogenic properties combined with cytotoxicity. By simply inserting a methylene bridge between 15-member carbocycle and norspermidine motif (**14**, Figure 7), the antimigration potency was doubled (38.4% vs. 20.3% inhibition at 0.6 µM) and cytotoxicity dramatically reduced (IC_50_ = 82.9 µM vs. 2.90 µM). Particularly, the enhanced antimetastatic activity was confirmed in vivo in terms of incidence and size of micrometastasis in the liver from pancreatic tumor. From experimental evidence on a series of polyamine-macrocycle derivatives emerged the importance of both functional groups for the activity and the perfect balance achieved with a methylene linker between them, while increasing the linker length reduced cellular viability and antimetastatic activity. These beneficial properties partially account for the ability of these conjugates to modulate cellular ceramide and sphingomyelin pools and interfere with membrane stability [38,39]. Thanks to this peculiar mechanism of action, some motuporamine derivatives were also identified as promising antimicrobial agents [40].

Polyamine toxins are naturally occurring conjugates composed of different polyamines fused with lipophilic acid heads that have been recently identified also as antiproliferative agents [41]. Different analogues were synthesized, varying the polyamine architecture and lipophilic group with the aim of increasing anticancer properties in breast cellular lines; however, except for few compounds that maintained micromolar potency, such as Spd-bearing **16** (Figure 7, IC_50_ = 3.15–12.6 µM), all other modifications caused a drop in activity. The most promising polyamine toxin remained natural **15** (Figure 7), with a sub-micromolar antiproliferative effect (IC_50_ = 0.55–3.31 µM) and selectivity over normal epithelial mammary cells (IC_50_ = 184.14 µM) [41].

### 2.2. Naphthalimides and Derivatives

Polycyclic cores such as naphthalimides have attracted particular attention in anticancer drug discovery programs for their peculiar abilities as DNA intercalating agents and tumor growth and metastasis suppressors [42]. Interestingly, some of them, such as amonafide, mitonafide and elinafide, have reached clinical trials as potential treatments for different types of cancers [43]. To achieve cell tumor specificity and enhance DNA binding, several naphthalimide–polyamine conjugates were developed, representing the leading topic for many years of medicinal chemistry research. All of those efforts were widely reported in 2013 in a review by Kelly and colleagues [43]; therefore, herein, we focus only on subsequent developments in the field.

**Figure 8:**
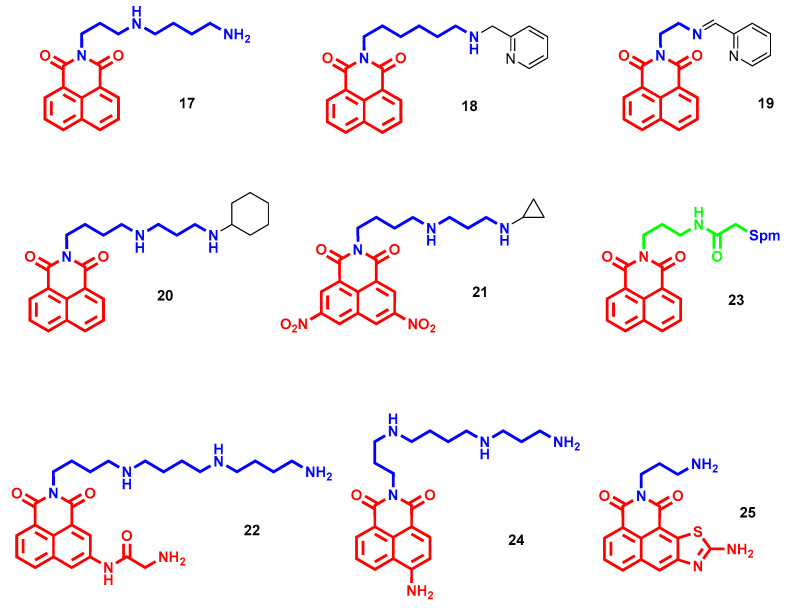
Naphthalimide–polyamine conjugates. The bioactive core is highlighted in red, the polyamine chain in blue, and the linker portion in green.

Firstly, polyamines of all lengths and distances among nitrogen atoms were attached to a naphthalimide core to study its effect on conjugates/DNA interaction and further rationalize their cytotoxic profiles [44,45]. Spectroscopic analyses confirmed that aromatic nuclei intercalate with DNA base pairs and polyamine motifs locate along grooves, mainly minor, and by increasing the number of nitrogen atoms or length, also enhance the binding constant of DNA–ligand complex [45]. One of the more interesting compounds for potency and selectivity over non-cancer cells (**17**, Figure 8) confirmed that through the abovementioned DNA engagement, it exerts an antiproliferative effect by arresting cells in G_2_/M phase and induces apoptosis in a dose-dependent manner [44].

In many experimental works, it has been reported that terminal-substituted polyamines can increase anticancer efficacy. In this context, Seliga and coworkers developed a series of polyamines with a pyridine head linked to naphthalimide and evaluated their anticancer properties [46]. The most potent compounds (**18** and **19** in Figure 8), albeit with completely different tethers, exhibited comparable IC_50_ values between 5.67 and 11.02 µM against human leukemia, breast and lung adenocarcinoma but not cervical cell lines. Both **18** and **19** demonstrated the ability to trigger apoptosis by inducing G_0_/G_1_ and G_2_/M arrest, respectively. Further investigations on **18** highlighted a lack of efficacy as an intercalator but possible activity as a minor groove binder [46].

Among different terminal alkyl heads tested in longer polyamine chains, the naphthalimide–polyamine conjugate **20** (Figure 8) with a terminal cyclohexyl head achieved the highest proliferation inhibition efficacy in several colorectal and hepatoma cell lines [47]. Particularly, by triggering ROS production and mitochondrial dysfunction, **20** induced p53-mediated apoptosis and migration suppression in hepatocellular carcinoma cells. Furthermore, at 15 mg/kg, it reduced hepatoma xenograft in mice by 50.34% in weight (vs. 44.17% with amonafide 5 mg/kg) and reduced pulmonary metastasis (61.80% vs. 41.24% with amonafide) without any toxic effects [47]. On the other hand, in dinitro or diamino naphthalimide conjugates, with the same Spd linker, the terminal cyclopropyl head was preferred for the anticancer efficacy, and nitro derivatives were more potent than amino ones [48]. The hit compound **21** (Figure 8) showed sub-micromolar cytotoxicity in hepatoma cells (even in a cisplatin-resistant line) paired to selectivity over normal liver cells and potent tumor growth inhibition at 5 mg/kg. Interestingly, **21** not only induced apoptosis through p53 upregulation, but also promoted cellular polyamine metabolism, thus disadvantaging rapid tumor growth [48]. In another work, from Xie et al., amidonaphthalimide was selected as a template to investigate the effect of polyamine chain length on the antitumor efficacy of naphthalimide–polyamine conjugates [49]. The 4,4,4 unsubstituted triamine tail of **22** (Figure 8) proved to be the most efficient substitution for selective cytotoxicity, especially in hepatic carcinoma cells (IC_50_ = 1.32 µM in HepG2 and IC_50_ = 0.98 µM in Huh-7). The remarkable in vivo antitumor (76.01% suppression at 5 mg/kg) and antimetastatic (75.02% reduction at 5 mg/kg) activities of **22** were then explained by a multifaceted mechanism of action: induction of DNA damage and apoptosis to kill cancer cells and lysosome-targeting modulation of polyamine catabolism (mainly downregulating SSAT and PAO) and autophagy to reduce metastasis formation [49]. An amido linker between Spm and unsubstituted naphthalimide gave **23** (Figure 8) with antitumor (70.92% tumor growth inhibition) and antimetastatic (62.42% lung metastasis inhibition) effects similar to those of **22** in mice hepatoma transplant models at lower dosage (1 mg/kg), highlighting the importance of a tailored polyamine tether. In this case, the proliferation inhibition of **23** involved a dose-dependent apoptosis induction triggered by mitochondrial impairment and ROS production [50]. Conversely, the antiproliferative effect of a similar compound (**24**, Figure 8), a 3-aminonaphthalimide directly fused with Spm, depends on hepatoma-selective induced apoptosis through the PI3K/Akt signal pathway. Particularly, the inactivation of serine/threonine kinase Akt **24**-related induced G_0_/G_1_ cell cycle arrest, mitochondrial dysfunction and caspase activation with consequent cell apoptosis [51]. Finally, an aminothiazole-fused naphthalimide with short polyamine (**25**, Figure 8), albeit moderately potent in cancer cell lines and with anti-hepatocellular carcinoma effects in vivo (52.63% inhibition at 5 mg/kg), represented one of the most efficient antimetastatic agents for pulmonary metastasis (75.73% inhibition at 5 mg/kg) mainly by upregulating E-cadherin and attenuating α6 integrin expression [52].

**Figure 9:**
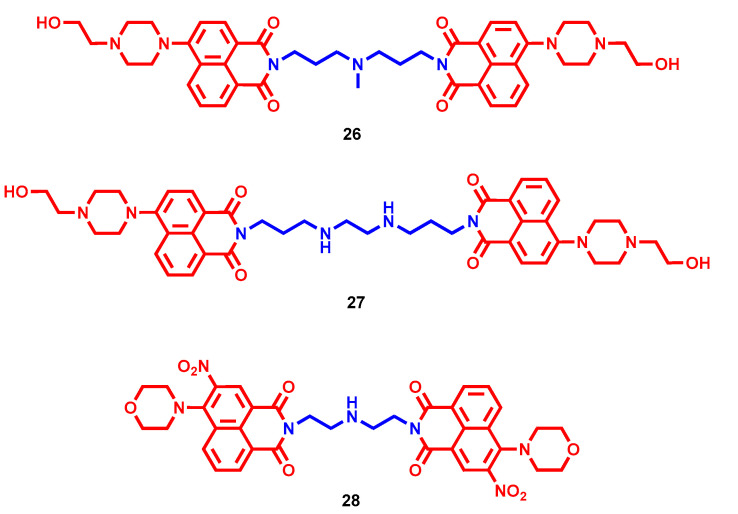
Bis-naphthalimide–polyamine conjugate. The bioactive core is highlighted in red and the polyamine chain in blue.

Several bis-intercalators with polyamine linker have been reported, showing higher DNA affinity than single fragments and a promising anticancer profile [53]. Therefore, the same approach was exploited using two substituted naphthalimide moieties bridged through *N*,*N*-bis(3-aminopropyl)methylamine. 1-Piperazinethanol substitution in 4-position emerged as preferred for cytotoxicity in different cancer cell lines (**26**, Figure 9). Particularly, bis-naphthalimide **26** triggered apoptosis and was confirmed to act as intercalator into the DNA [54]. Interestingly, also in a series of bis-naphthalimides connected with a *N*,*N*-bis(3-aminopropyl)ethylenediamine linker, the piperazine ethanol derivative resulted in one of the best cytotoxic compounds (**27**, Figure 9), with an IC_50_ ranging from 1.60 ± 0.37 µM in MGC-803 to 2.73 ± 0.18 µM in HeLa cancer cell lines. Differently from **26**, it showed weak intercalator properties paired to strong binding interactions with DNA helix, which resulted in a promising bioimaging tool [55]. In a diethylenetriamine-bridged series, the 3-nitro-4-morpholino analogue (**28**, Figure 9) was highlighted by low micromolar antiproliferative activities in human ovarian, bladder, gastric and nasopharyngeal cancer cell lines. In an in vivo xenograft cancer model, **28** reduced the tumor weight by 44.7% at 4 mg/kg after 21 days of treatment, which was higher than mitonafide and its mono-naphthalimide analogue at the same concentration (34.2% and 34.4%, respectively). Further investigations at the cellular level demonstrated that induced apoptosis, DNA intercalation and cell cycle arrest could account for **28**’s cytotoxic efficacy [56].

**Figure 10:**
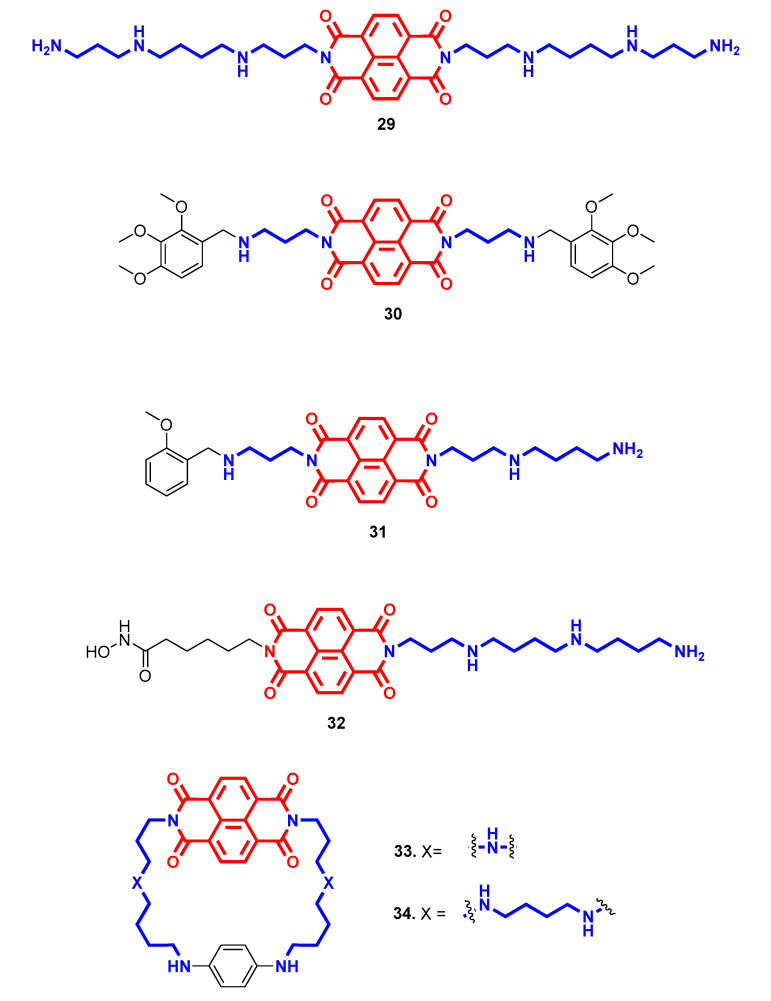
Naphthalene diimide–polyamine conjugates. The bioactive core is highlighted in red and the polyamine chain in blue.

Naphthalene diimides (NDI) constitute naphthalimide derivatives with well-known anticancer properties, mainly acting as intercalator agents or binders to non-canonical DNA structures. Notably, they have been reported as more active than their monoimide or biphenyl diimide analogues in this respect [57,58]. The Spm derivative **29** (Figure 10) resulted as the most potent antiproliferative agent among all of the polyamine-attached unsubstituted naphthalene diimides. Iv-administered 0.2 mg/kg **29** in mice hepatoma transplant models increased the lifespan by 2.3-fold, by inhibiting tumor growth and primarily reducing tumor metastasis without any systemic toxicity, unlike amonafide. In this case, preliminary results indicated apoptosis induction in a ROS-mediated mitochondrial pathway as the mechanism of action for **29** [58]. The lengths of the two basic side chains and terminal substituted benzyl heads were also evaluated for the naphthalene diimide core’s antiproliferative profile [59]. 2,3,4-Trimethoxy benzyl derivative **30** (Figure 10) demonstrated remarkable cytotoxicity in several cancer cell lines with a sub-micromolar profile by prompting caspase activation and apoptosis. Interestingly, it exerted a multifaced profile with different putative interactions with different DNA structures, accounting for its biological profile: intercalator only with dsDNA and “sandwich-type” stacking for G-quadruplex (G4) DNA conformation [59]. A stable lyophilized liposomal formulation of **30** was then developed to facilitate potential iv anticancer treatment, maintaining a similar in vitro cytotoxic profile after 72 h incubation [60]. To increase the selectivity for G4, the DNA substructure more prevalent in tumor tissues and usually located in the promoter region of oncogenes, asymmetric NDIs were developed with substituted benzylpropylendiamine in one arm and different polyamines in the other [61]. Compound **31** (Figure 10) with an Spd chain resulted as the most potent and selective binder for G4 over dsDNA (ΔTm = 29 °C vs. 12.2 °C at 2.5 µM, respectively), paired with an in vitro sub-micromolar anticancer profile. The moderate inhibition of two DNA processing enzymes such as topoisomerase IIα and TAQ-polymerase was attributed to DNA–ligand interaction, with a key role played by the polyamine tail rather than the ligand/protein one [61]. In a multitarget approach, the same scaffold was further exploited to add to DNA binding abilities the histone deacetylase (HDAC) inhibitory properties with the aim of achieving polyhedral anticancer efficacy [62]. Interestingly, it was found that compounds **30** and **31** impaired the growth of metastatic castration-resistant prostate cancer (mCRPC), a lethal form of prostate cancer, thanks to their ability to target and rearrange into a G4 a region within the promoter of epidermal growth factor receptor (EGFR), reducing the receptor production [63].

By substituting in one side a benzylamine tail with an alkyl hydroxamic acid functional group inspired by Scriptaid, which represents a naphthalimide-based HDAC inhibitor, different polyamines were tested in the other branch to reach both targets. Once again, the spermine homolog derivative **32** (Figure 10) joined the best DNA binding, both ds and G4, with HDAC inhibitory capabilities, with a preference for isoform 6, which resulted in a micromolar antiproliferative effect. Furthermore, the simultaneous interaction with DNA and HDACs provided **32** with a peculiar cell phenotype reprogramming property that prompted cancer cells toward a less aggressive and migratory profile through a reduced conversion from epithelial to mesenchymal phenotype [62].

In another attempt to increase G4 affinity, macrocyclic NDIs have been created by locking side polyamine chains through a phenyl ring [64]. The efficiency and selectivity in G4 binding directly correlates with the length of the polyamine chain and number of nitrogen atoms therein, with Spm derivative **34** (Figure 10) as the best of the series (ΔTm = 26.8 °C at 1 µM). On the contrary, the in vitro anticancer profile was inversely related to G4 affinity, where the less stabilizing agent **33** (Figure 10) with shortest chain exerted the highest antiproliferative efficacy. Surprisingly, an analogue bearing an ether side junction with almost no DNA-binding ability exerted cytotoxicity as the most potent polyamine derivative, highlighting criticisms in in vitro-to-cell translation, probably due to unfavorable physico-chemical properties of macrocyclic ligands [64].

**Figure 11:**
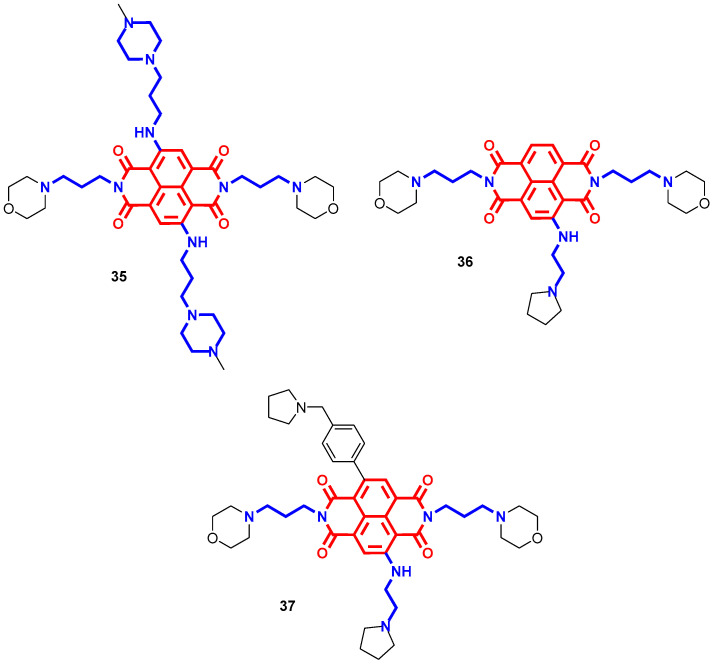
Polysubstituted naphthalene diimide–polyamine conjugates. The bioactive core is highlighted in red and the polyamine chain in blue.

Prompted by these encouraging results, researchers further attempted tri- or tetrasubstitution on a naphthalene diimide core with polyamine tails to boost the DNA affinity and antiproliferative effects [65]. Figure 11 shows the most potent polysubstituted naphthalene diimides developed by Neidle’s group, with remarkable antitumor activities in pancreatic cancer animal models and cell lines [66,67,68]. They are all characterized by heterocycle end-groups with tertiary amines, to increase their basicity, which resulted in higher DNA affinity and more potent G4 binders and stabilizers. Particularly, the terminal physiologically protonated nitrogen atoms drove the interaction with DNA phosphate backbone, while the morpholino groups mitigated the overall pharmacokinetic properties and maximized the binding onto G4 substructures. Differences in polyamine side chains contributed to the compounds' G4 selectivity, while all retained selectivity over DNA duplex [69]. Symmetrically tetrasubstituted **35** (Figure 11) demonstrated the ability to potently bind different promoter or telomeric quadruplexes and induced cellular senescence, leading to a potent anticancer profile in vivo (80% decrease in tumor growth after 40 days with 12 iv-administered doses of 15 mg/kg) thanks to massive tumor uptake [66,70]. The trisubstituted **36** (Figure 11) increased the antiproliferative efficacy of **35** toward pancreatic ductal adenocarcinoma by reducing tumor volume by 73% at 15 mg/kg after 28 days of treatment (vs. 66.7% with **35** or gemcitabine). Furthermore, this efficiency partially accounted for **36**–quadruplexes binding, which resulted in potent down-regulation of several genes involved in tumor survival, metastasis and gemcitabine resistance paired with increased DNA damage [67,71]. Further structural optimizations on the same core led to the asymmetrically tetrasubstituted **37** (Figure 11) with enhanced quadruplex affinity and cytotoxicity. Furthermore, **37** overcame tumor regrowth after the end of 28 days of treatment with **36**, resulting in even more potent effects at a lower dose (86.6% tumor volume reduction at 1 mg/kg vs. 73.3% at 15 mg/kg) [68,69].

### 2.3. Miscellaneous

**Figure 12:**
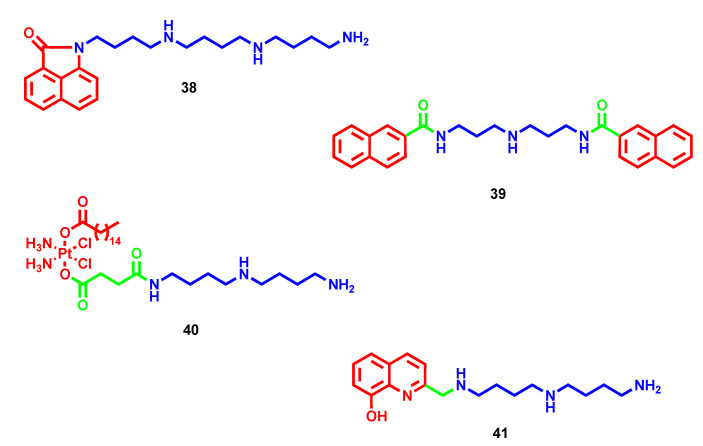
Other polyamine conjugates with antiproliferative properties. The bioactive core is highlighted in red, the polyamine chain in blue, and the linker portion in green.

Besides naphthalimides and NDIs, several other scaffolds endowed with anticancer properties were attached to polyamines to further optimize and boost their efficacy. Inspired by the first one, smaller benzo[*cd*]indol-2(1*H*)-one cores were differently substituted or attached to polyamines for verifying if the promising antiproliferative effect of naphthalimide–polyamine conjugates was retained [72]. Of particular note, the unsubstituted homospermine hybrid **38** (Figure 12) constituted a potent antimetastatic agent (82.5% inhibition after 15 days of 1 mg/kg treatment) and a moderate antitumor agent (46.9% inhibition after 15 days of 1 mg/kg treatment), while other substitutions on the benzo[*cd*]indol-2(1*H*)-one scaffold led to low anticancer profiles in vitro. By partially entering through PTS, **38** taken up in lysosomes triggered polyamine catabolism and caspases activation to reduce cell migration and induce apoptosis/autophagy-mediated cytotoxicity [72].

In order to achieve greater DNA affinity and selectivity, bis-intercalators have been developed by inserting different polyamine linkers between two intercalating functional groups such as quinazoline, quinoline, naphthalene, indole, coumarin and chromone [73,74,75]. All of the reported compounds showed low cytotoxicity with IC_50_ above 10 µM, a concentration at which it is difficult to gain selectivity over non-cancer cells, except for bis-napthalene derivatives such as **39** (Figure 12). In this case, it demonstrated an IC_50_ of 7.63 µM and 6 µM in prostate carcinoma and mammary gland adenocarcinoma cell lines, respectively, thanks to an observed mild ability to stabilize double helix DNA through stacking interactions [73].

Albeit widely used in clinical treatments, platinum-containing antineoplastics, as well as other alkylating agents, have prominent side effects due to low selectivity that remain their major issues. In an attempt to increase the antimetastatic efficacy and tumor-specific targeting, new platinum-polyamine complexes have been recently developed [76]. In this case, the new conjugates were far more potent in comparison to parent drugs where cisplatin and unsubstituted polyamine derivatives were preferred over oxaliplatin or substituted analogues, with **40** (Figure 12) emerging as a hit. In particular, a remarkable antimetastatic effect was verified for the homospermidine hybrid **40** due to upregulation of polyamine catabolism and ROS that reduce polyamine content and discourage cell migration as well as overcome cisplatin resistance. Furthermore, exploiting selective uptake through PTS in cancer tissue, it triggered p53-mediated apoptosis and platinum-induced DNA damage that resulted in notable antitumor activity in a mouse breast cancer model (75.44% inhibition at 20 mg/kg after 13 days of treatment) compared to cisplatin (57.06% inhibition at 5 mg/kg) and without maintaining its toxicological profile [76].

Metal-sequestering agents have been widely considered as potential anticancer treatments thanks to the correlation between metal dyshomeostasis, commonly upregulation, and tumor environment. Metals such as iron, copper and zinc are cofactors of several enzymes and essential for physiological processes such as survival, growth and proliferation; therefore, their depletion in fast-growing cells has been proposed as a tumor therapy. Depending on the architecture, polyamines hold chelating properties paired with tumor-driving force, making them suitable multifunctional ligands for anticancer research [77]. In particular, some polyamine-based theranostic agents were developed by merging optical imaging to iron-chelating tumor-targeted cytotoxic properties [78,79]. In addition to macrocyclic polyamines exploited mainly for radiometals, even linear polyamines bear chelating properties that were amplified when they were grafted to other chelating fragments (e.g., hydroxyquinoline motif). Particularly, conjugate **41** (Figure 12) with a homospermidine tail exploited a selective PTS-mediated uptake to direct its dose-dependent cytotoxic effect (IC_50_ of 1.4 µM in CHO cells). In this case, the polyamine moiety played a double role: as vector and for boosting the hydroxyquinoline iron chelating capacity [80].

## 3. Antimicrobial Agents

Polyamines are polycationic molecules ubiquitously expressed in nature. Some of them have been proved to exert a critical influence on microorganism metabolism and proliferation, and accordingly were considered as potential starting points for antimicrobial drug development. The involvement of polyamines in cellular machinery, and then the biological activity of polyamine derivatives, depend closely on the family of pathogens involved. Therefore, herein, the polyamine conjugates are handled according to the targeting microorganism.

### 3.1. Antibiotics

In bacteria, besides core physiological functions, polyamines proved essential for their pathogenesis by optimizing the interplay between host cells and infecting bacteria [81]. Furthermore, discrepant outcomes were reported regarding polyamines’ influence on antibiotic activity. In some cases, polyamines seem to induce resistance by modulating outer membrane permeability [82,83], while in other cases, co-treatment with exogenous polyamines can increase the antibiotic susceptibility of some strains of both Gram-positive and Gram-negative bacteria [84]. These premises, along with the discovery of the strong and wide antibiotic effects of natural aminosterols (e.g., squalamine and trodusquemine) bearing Spd or Spm moieties, have represented the starting point for antibacterial polyamine conjugate development [85]. Furthermore, polycationic amine tail conjugation has been validated as a medicinal chemistry strategy to re-empower the efficacy of antibiotic agents against resistant strains [86,87]. Following the same path of squalamine, other bioactive sterols, but with modest or no bacteriostatic activity, were linked to polyamines and their antimicrobial profiles evaluated. Moreover, these designed cationic amphiphilic molecules were considered to be biomimetics of endogenous peptide antibiotics [88].

**Figure 13:**
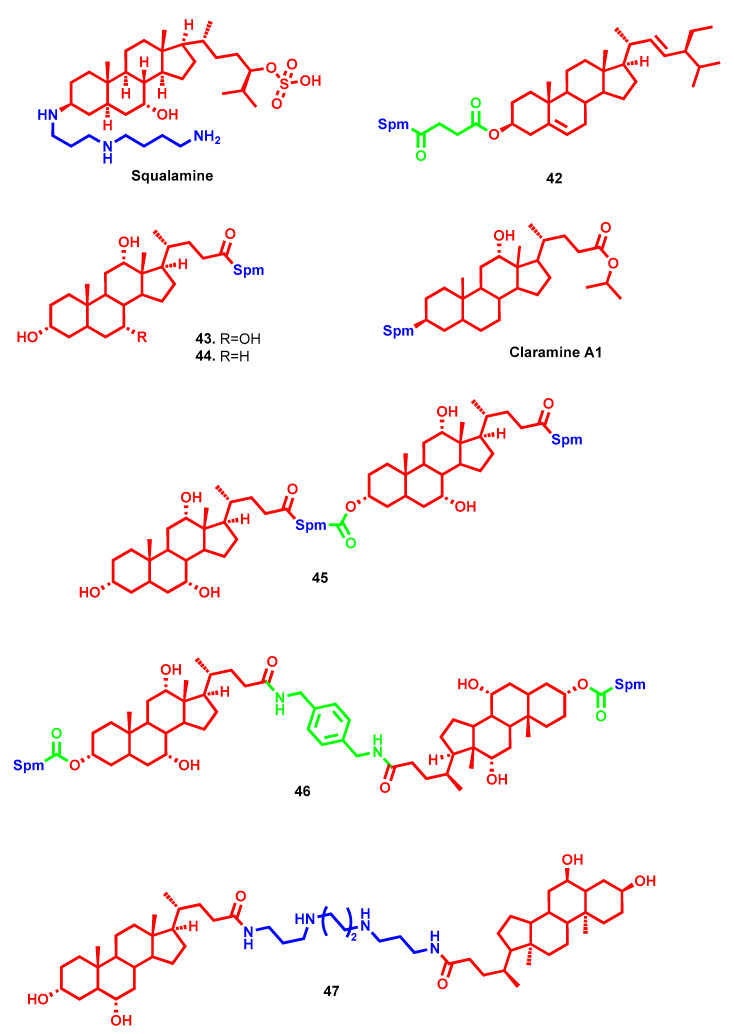
Sterol–polyamine conjugates with promising antimicrobial profiles (part I). The bioactive core is highlighted in red, the polyamine chain in blue, and the linker portion in green.

**Figure 14:**
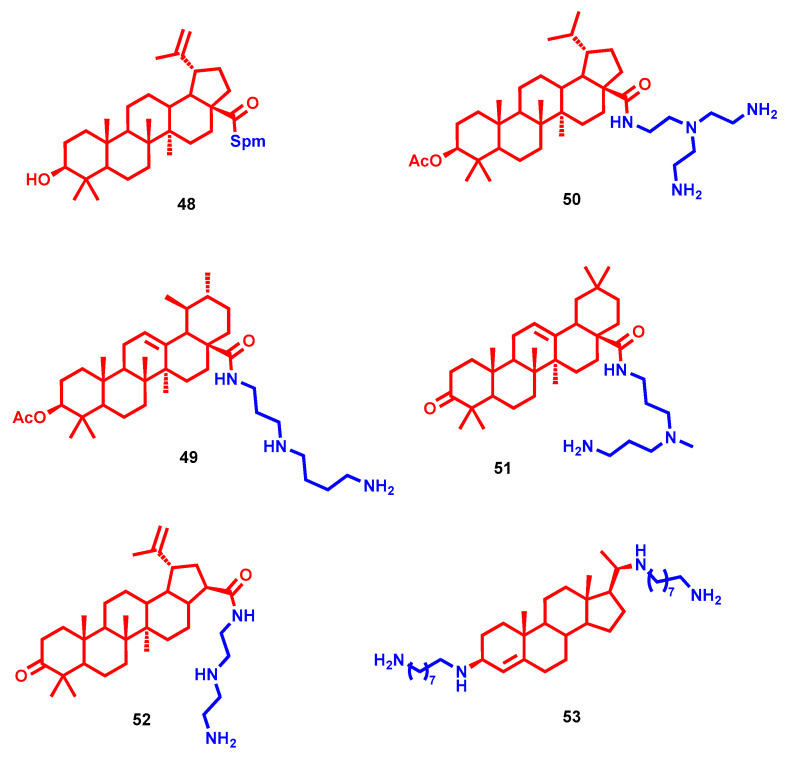
Sterol–polyamine conjugates with promising antimicrobial profiles (part II). The bioactive core is highlighted in red and the polyamine chain in blue.

One example is represented by the hybrid **42** (Figure 13), which originated from the linkage of a phytosterol (i.e., stigmasterol) with Spm, showing a selective inhibition effect on *S. aureus* and reducing bacterial growth to 25% within 12 h at a concentration of 50 µg/mL [37]. Similarly, several other sterol backbones were exploited for polyamine conjugation, such as cholic or deoxycholic acid (compounds **43**, **44** and **45**, Figure 13), betulinic acid and β-sitosterol (compounds **42**, **46** and **50**, Figure 13 and Figure 14) or ursolic acid (compound **13**, Figure 6). Analogs of squalamine but constituted of cholic (or deoxycholic) acid and Spm showed significant, wide and non-selective antibacterial activities toward both Gram-positive and Gram-negative bacteria (compounds **43** and **44**, Figure 13). On the other hand, their head-to-tail dimeric conjugates exerted strong antibiotic efficacies against a broad spectrum of Gram-positive bacteria, ranging from *Enterococcus* to *Staphylococcus* and *Streptococcus* with MICs in the low µM profile. In particular, cholic acid dimer **45** (Figure 13) highlighted the highest activities, similarly to squalamine, whereas the deoxycholic conjugate was the most potent among monomeric analogs [89]. Additionally, tail-to-tail cholic acid Spm conjugates were reported linked with different tethers. In compound **46** (Figure 13), the rigid bridge led to an increased anionophoric efficiency that accounted for their antibacterial activity [90]. More recently, a deoxycholic derivative with Spm moiety directly attached at C3 was developed and called Claramine A1 (Figure 13) [91]. It demonstrates antimicrobial activities against a large panel of both Gram-positive and Gram-negative bacteria, including multi-drug resistant pathogens, with MIC values ranging from 2 to 32 μg/mL and a multifaceted mechanism of action dependent on the type of strain. In Gram-positive bacteria, Claramine A1 can disrupt membrane integrity via depolarization, whereas in Gram-negative strains, it influences cell membrane permeabilization by altering proton homeostasis, in addition to possessing synergistic effects [91]. The same tail-to-tail approach was recently evaluated in different polyamine conjugates linked through a C-24 amide functional group by exploring different cholic acid head groups. In this case, the hyodeoxycholic acid analogue **47** (Figure 13) exhibited remarkable Gram-positive antibacterial (MIC ≤ 0.20 µM in *S. aureus* strains) and antifungal activity (MIC ≤ 0.20 µM in *C. albicans* and MIC = 0.80 µM in *C. neoformans*) and was devoid of any cytotoxic or hemolytic effects at the top dose tested (32 µg/mL). The bactericidal properties of **47** were also confirmed in several bacterial strains, albeit its exact mechanism of action has still to be elucidated, while membrane perturbation/ATP depletion and antibiotic enhancement have been ruled out [92].

The two betulinic acid-based Spm hybrids **48** (Figure 14) and **10** (Figure 6) demonstrated high and selective antimicrobial activity [31]. Compound **48**, bearing conjugation of the two moieties through a carboxy functional group at C28, displayed activities toward *S. aureus* (MIC of 12.5 µg/mL) and *E. coli* (MIC of 6.25 µg/mL), while **10**, with an hemisuccinate bridge at C3 between the two fragments, was more active against *S. aureus* and *E. faecalis* (MIC of 3.125 µg/mL for both). Both of them showed weak or no activity toward Gram-negative bacteria [31]. Triterpenoids alone, like previously reported sterols exploited as anchor points for polyamine conjugation, exert modest bacteriostatic activities, whereas their amine hybrids show considerable increases in antimicrobial activity. 3-Acetylated betulinic, ursolic and oleanolic derivatives were conjugated at C28 with different polyamines or guanidines, and their antimicrobial activities were evaluated toward different strains [93]. The ursolic **49** and betulinic **50** derivatives, bearing biogenic Spd and tris(2-aminoethyl)amine, respectively, were among the best of the series in terms of their anti-staphylococcal potential (MICs ≤ 0.25 µg/mL), with an antibacterial effect superior to that of clinically used vancomycin (MIC = 1 µg/mL). In parallel, their antifungal activities were also evaluated against *Cryptococcus neoformans*, revealing them to be 65 times more potent than the drug fluconazole. These findings, together with low toxicity in mammalian cells, confirmed once more the antimicrobial potential of polyamine–sterol conjugates, which would pave the way for further clinical investigations [93].

Oleanonic acid represents another bioactive terpene that has been exploited for conjugation with diamines or polyamines to evaluate their profiles as antibiotic potency enhancers [94]. Compound **51** (Figure 14) with *N*-methyl-norspermidine bridged through C17 carboxamide functional group was shown to possess low MICs (6.25–25 µM) against a wide panel of strains, particularly some multidrug-resistant bacteria (e.g., *P. aeruginosa* CIP100720 and *K. aerogenes* EA289). Interestingly, the mechanism of action accounted for the antibiotic activity of **51** in *P. aeruginosa* PA01 cells through the disruption of the outer bacterial membrane [94]. Furthermore, as proof of their polyhedral profile, an amidoethylpiperazine derivative of oleanonic acid recently demonstrated moderate antiviral activity [95].

Heterobetulonic acid and ursolic acid were attached at C17 with Spm by carboxamide functional group (**12** and **13**, Figure 6), and their MIC values were 6.25 µM for *Staphylococcus aureus*, *Streptococcus mutans* and *Listeria monocytogenes*, identically for both compounds and for all three microorganisms [33]. Another betulonic acid-diethylentriamine conjugate, compound **52** (Figure 14) showed partial activity against methicillin-resistant *S. aureus* and the yeast *C. neoformans*, exerting growth inhibition of 71.80% and 62.56% at 32 µg/mL, respectively [32].

To increase the hydrophilicity and cationic charge, a series of bis(polyamino)steroid derivatives were reported with amino tails attached at C3 and C20 [96]. Interestingly, the chain length has a major impact on the antimicrobial activities, pointing to eight carbon atom the optimal length in this respect (**53**, Figure 14). Compound **53** showed potent anti-staphylococcal activity and moderate to excellent antibacterial potency against Gram-negative *E. coli* (MIC = 5 µg/mL) and *P. aeruginosa* (MIC = 2.5–10 µg/mL), while low or no activity was shown against *I. limosus* and *B. cepacia*. Particularly, it showed direct and fast bactericidal effects against Gram-positive *S. aureus*, also acting through membrane depolarization. On the other hand, disruption of the outer membrane, similarly to colistin with a detergent-like mechanism, accounted for its Gram-negative antibacterial effect [96].

**Figure 15:**
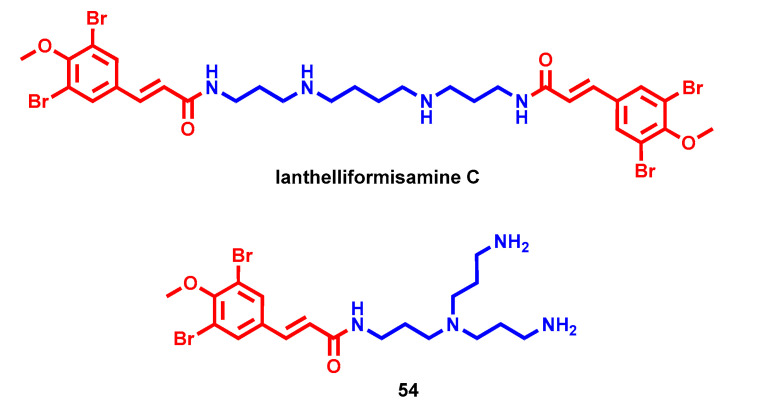
Sponge-derived polyamine conjugates. The bioactive core is highlighted in red and the polyamine chain in blue.

In 2012, Xu et al. identified three bromotyrosine-derived metabolites from the sponge *S. ianthelliformis* equipped with antibacterial activity [97]. Particularly, ianthelliformisamine C, bearing an Spm linker between two substituted cinnamic functional groups, showed MICs from 12.5 to 25 µg/mL against different Gram-positive and Gram-negative bacterial strains [98]. Moreover, ianthelliformisamine C and its synthetic analogue **54** featuring a tris(3-aminopropylamine) chain (Figure 15) were demonstrated to restore doxycycline activity against several Gram-negative strains at low micromolar concentrations. In the case of *P. aeruginosa* strains, besides doxycycline, they proved to improve even chloramphenicol (at 12.5 and 100 µg/mL, respectively) and cefepime activities (at 1.6 and 0.4 µg/mL, respectively) without any cytotoxicity issues until >200 µg/mL. Further experimental investigations suggested a possible modulation of drug transporters, accounting for the antibiotic susceptibility of compound **54** [98].

**Figure 16:**
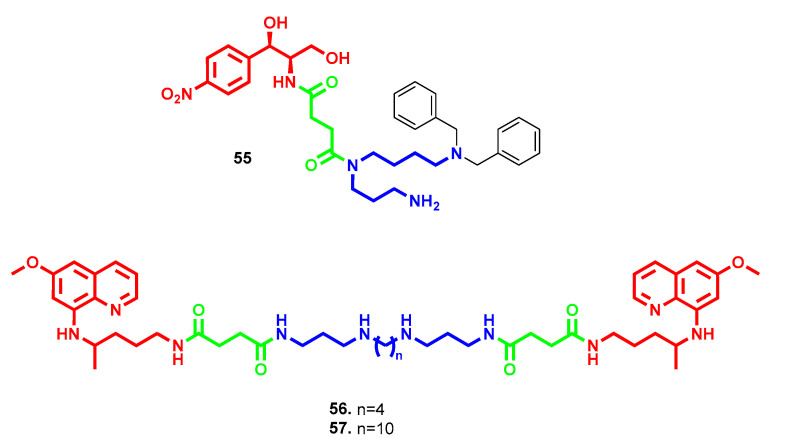
Antimicrobial drug–polyamine conjugates. The bioactive core is highlighted in red, the polyamine chain in blue, and the linker portion in green.

Following the strategy of polyamine conjugation to tackle antibiotic resistance, even some approved antimicrobial drugs were exploited as attaching sites. Chloramphenicol is a broad-spectrum antibiotic that acts by inhibiting protein synthesis, but its use is limited due to its adverse effects. Kostopoulou et al. developed a series of chloramphenicol–polyamine derivatives with different polyamine architectures and docking sites with the aim of improving chloramphenicol’s activity and uptake [99]. The most potent conjugate, compound **55** (Figure 16), possesses *N8*, *N8*-dibenzylspermidine attached through *N4* to a succinate bridge, in replacement of the dichloroacetyl group of chloramphenicol. It exerted comparable or improved antibacterial potency against *S. aureus* (IC_50_ = 4.7 µM vs. 3.1 µM) and *E. coli* (IC_50_ = 9.4 µM vs. 6.2 µM), particularly against resistant *E. coli* strains (IC_50_ = 9.4 µM vs. 15.5 and 24.7 µM), with reduced toxicity against human health cells. Furthermore, the same mechanism of action of chloramphenicol was maintained in compound **55**, where the precursor scaffold competes with aminoacyl-tRNA binding to ribosome A-site, while the polyamine chain could interfere with the rotation of aminoacyl-tRNA toward the P-site [99]. Analogously, the antimalarial primaquine was exploited as a bioactive head and conjugated with different polyamines, through a succinyl linker, to explore their antimicrobial and antibiotic adjuvant properties [100]. Compound **57** (Figure 16) with a decyl central tether emerged as the most potent antimicrobial of the series, with selectivity against *S. aureus* (MIC = 3.3 µM) and the yeast *C. neoformans* (MIC = 1.7 µM). Interestingly, compound **56** (Figure 16), with an Spm linker that was devoid of any antimicrobial activity, showed enhanced doxycycline activity against *P. aeruginosa* equipotent to that of **57** (MIC = 6.25 µM for potentiation of 2 µg/mL doxycycline) coupled with more modest potentiation toward *E. coli* (MIC = 50 and 12.5 µM, respectively) [100].

### 3.2. Antiprotozoa

**Figure 17:**
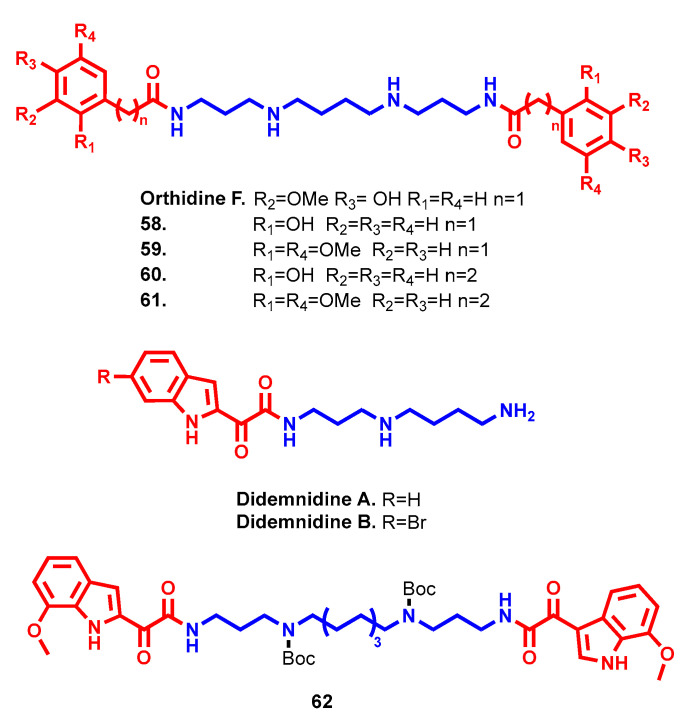
Marine-derived polyamine conjugates. The bioactive core is highlighted in red and the polyamine chain in blue.

Besides bacteria (and fungi in some cases), several polyamine conjugates were investigated for their antimicrobial profiles, mainly related to different protozoa such as kinetoplastids (e.g., *Trypanosoma brucei*, *Trypanosoma cruzi* and *Leishmania donovani*) and *Plasmodium falciparum*. Particularly, their development was built on the essential role played by polyamines in parasitic cellular machinery, and thus exploited it to direct the antiprotozoal efficacy of polyamine conjugates [101]. Two of the first polyamine antimalarial hits derived from a screening on marine natural products afforded orthidine F (IC_50_ = 0.89 µM) and didemnidine A and B (IC_50_ = 41 µM and 15 µM, respectively) [102,103]. Furthermore, orthidine F, unlike didemnidines, showed promising selectivity while avoiding any cytotoxicity against a mammalian cell line, serving as a suitable starting point in antimalarial drug design (Figure 17). Different polyamine linkers and substituents on arylacetic heads were evaluated with the aim of increasing potency against *P. falciparum* strains while maintaining good selectivity. Spm analogues, like the parent compound, retained the best antimalarial profile, with increased potency relative to orthidine F. Regarding aryl substituents, 2-hydroxy derivative (compound **58**, Figure 17) achieved the highest potency (IC_50_ = 8.6 nM), which was not preserved in other 3- and 4-isomers associated with the original non-cytotoxic profile. Of note, 2,5-disubstituted analogue (compound **59**, Figure 17) also exerted interesting antimalarial activity (IC_50_ = 19 nM), albeit accompanied by higher cytotoxicity (IC_50_ = 88 µM) [102]. By reducing the distance between aryl and amide functional groups, a small reduction in activity with increased toxicity was verified, but increasing the distance yielded equipotent or more active 3-phenylpropanamide compounds **60** and **61** (Figure 17) while retaining good selectivity (IC_50_ = 15 nM, SI = 5700 and IC_50_ = 6.1 nM, SI = 16,230, respectively) [104]. Concurrently, by introducing longer polyamine chains, antimicrobial activity generally decreased (except for 2-hydroxysubstituted PA3-8-3, which achieved IC_50_ = 1.3 nM) and toxicity dramatically increased. Compounds **58** and **60** were also tested in vivo in *P. berghei* infected mice, revealing no increase in mean survival time but a 27.9% reduction in parasitemia for **62** when tested repeatedly at 30 mg/kg/day ip [104].

A similar structure–activity relationship study of polyamine linkers and capping indoles was conducted starting from didemnidine scaffolds to improve their modest antiprotozoal activities [105]. Starting with the experimental evidence for the higher antimalarial activities detected in bis-bromoindole analogues, a double-headed drug design strategy was conducted, such as occurred before with orthidine derivatives. An indolglyoxylamide cap was preferred over indolacetamide, pointing to 7-methoxy substitution as the preferred one. Surprisingly, compounds bearing Boc-protected amino groups of central tether demonstrated higher activity and selectivity over the non-protected analogues, while PA3-8-3 was identified as the optimal length in this respect. Compound **62** (IC_50_ = 92 nM, SI ≥ 1300, Figure 17), featuring all of the identified structural requirements, emerged as the best derivative of the series and was thus selected for in vivo studies. Ip administration to *P. berghei* infected mice for 4 days at 50 mg/kg/day led to 20.9% parasitemia reduction but without an increase in mean survival time [105]. Although the identified structural requirements to achieve optimal antimalarial potency for orthidine and didemnidine derivatives were slightly different, orthidine analogues generally confirmed the best antiprotozoal and toxicity profiles already encountered with their parent natural compounds.

**Figure 18:**
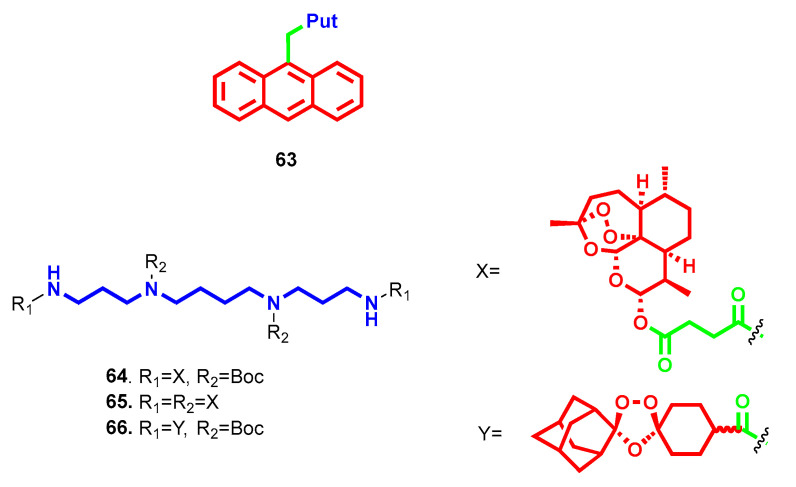
Antimalarial polyamine conjugates. The bioactive core is highlighted in red, the polyamine chain in blue, and the linker portion in green.

Differently from the natural polyamine-bearing compounds, some bioactive scaffolds endowed with antimalarial potency were conjugated with polyamines to leverage the delivery of these latter toward parasitic cells. A small set of polyamines were attached to the cytotoxic nucleus of anthracene, and the resulting hybrids were shown to be capable of inhibiting *P. falciparum*’s growth in human erythrocytes [106]. Particularly, Put derivative **63** (Figure 18) was the analogue with the highest antimalarial potency (IC_50_ = 0.64 ± 0.04 µM) and selectivity over a panel of human cancer cell lines, while for the others, IC_50_ and cytotoxicity against *P. falciparum* were comparable. As evidence of this, **63** was demonstrated to be selectively taken up in infected erythrocytes over uninfected ones and inhibited the parasite’s cell cycle within the first 24 h of exposure, in addition to inhibiting polyamine uptake by competing with the transport [106]. Similarly, the antimalarial drug artemisin and bioactive 1,2,4-trioxolanes were conjugated with different polyamines to evaluate if this structural modification led to the enhancement of biological activities [107,108]. Generally, the artesunate analogues were more potent than trioxolanes, and PA3-4-3 was found to be the best chain length, whereas with a longer tether, cytotoxicity issues arose independently of the type of substitution. Bis-(Boc)-bis-artesunate derivative **64** and (tetra)-artesunate conjugate **65** (Figure 18) emerged with the best antimalarial activities against the drug-sensitive *P. falciparum* NF54 strain (IC_50_ = 0.4 and 0.3 nM, respectively) and selectivity index over cytotoxicity in a rat cell line (SI = 30,250 and 37,333, respectively). In the trioxolane series, compound (bis)-Boc-protected **66** (Figure 18) turned out to be the most efficient against *P. falciparum* NF54 (IC_50_ = 5.1 nM), with low cytotoxicity (IC_50_ = 65.85 nM). In *P. berghei* infected mice, only **64** and **65** reduced parasitemia (99.8% and 95.5%, respectively) with 30 day survival rates, while trioxolanes turned out to be ineffective. In this case, polyamine conjugation with artesunate retained the promising biological activities of the parent compound, whereas trioxolane acid remained more potent relative to its derivatives [108].

**Figure 19:**
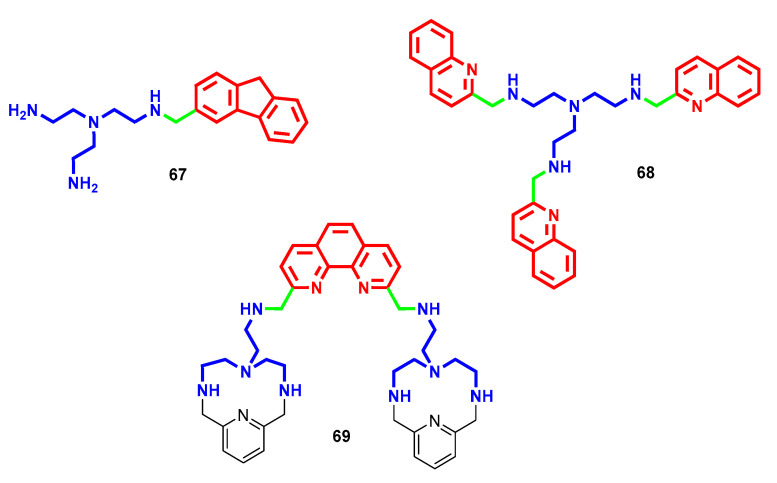
Antiketoplastid polyamine conjugates. The bioactive core is highlighted in red, the polyamine chain in blue, and the linker portion in green.

In kinetoplastids, the polyamine pathway is considered one of the main targets to tackle parasitemia because, apart from the essential role of polyamines in parasitic growth and survival, in some cases the parasite is not able to produce them by itself, and proper uptake from the host becomes vital [101]. For example, *T. cruzi* does not contain enzymes to synthesize de novo Put and Spm and, therefore, their intracellular availability relies only on transport processes. That explains why polyamine transporters are targeted to alter parasite viability [109]. Furthermore, Put and/or Spd are involved in trypanothione biosynthesis, the main parasite defense mechanism against oxidative stress [110]. Based on these, several polyamine derivatives have been synthesized over the years in the search for effective antikinetoplastid chemotherapeutics, and all of these efforts were properly described and reported in a review by Labruère et al. in 2017, to which the interested reader is referred [111]. More recently, a series of differently substituted tris(2-aminoethyl)amines were reported to bear trypanocidal effects against *T. cruzi*, identified as the etiological agent of Chagas disease [112]. Different aromatic caps attached at terminal amino groups were evaluated, and derivatives bearing monofluorene (compound **67**, Figure 19) or tris(2-quinoline) (compound **68**, Figure 19) substitutions yielded the best activity profile (in low micromolar range) against different *T. cruzi* strains and forms. Furthermore, both of them demonstrated a very low toxicity profile in a mammalian cell line (i.e., over 1500 mM). In infected mice, compound **68** demonstrated almost no activity, whereas compound **67** caused a drop of 72% in parasitemia by the 23rd day of treatment (comparable to the drug benznidazole) and was effective in both acute and chronic phases as well as preventing reinfection after immunosuppression. Investigations on their potential mechanism of action identified the inhibition of enzymes involved in the catabolic glucose pathway for **67** and induced alteration in mitochondrial membranes for **68** [112]. For compound **69** (Figure 19), redox-stress by inhibition of Fe-SOD enzyme and mitochondria-dependent bioenergetic collapse were considered the principal induced dysfunctions accounting for its trypanocidal effect. Furthermore, in infected mice after oral administration (i.e., 20 mg/kg·day for five consecutive days), **69** showed a ~65% reduction on the day of maximum parasitemia associated with an overall parasite burden and parasite load decrease, more efficiently than benznidazole [113].

Due to the essential role played by polyamine transporters in *T. cruzi*, several polyamine conjugates were tested to verify their inhibitory properties in this respect. Among all of the derivatives evaluated, **63** (Figure 18) emerged as the most interesting because it was able to join inhibition of Put and Spd transport (respectively IC_50_ = 5.02 ± 0.39 µM and IC_50_ = 8.78 ± 1.04 µM) to trypanocidal activity (IC_50_ = 16.97 ± 1.16 µM in epimastigote and IC_50_ = 0.46 ± 0.02 µM in trypomastigote) [109].

## 4. Antioxidant

**Figure 20:**
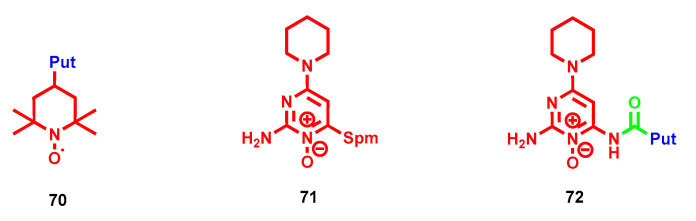
Antioxidant polyamine conjugates. The bioactive core is highlighted in red, the polyamine chain in blue, and the linker portion in green.

The correlation of polyamine cellular content and oxidative stress condition is well-known, and several experimental examples are already reported above. With the aim of improving their antioxidant efficacy, polyamines have been conjugated with other radical scavenger pharmacophores such as (2,2,6,6-tetramethylpiperidin-1-yl)oxyl (TEMPO). Particularly, TEMPO exerts a multifaceted antioxidant efficacy by inhibiting myeloperoxidase, a heme peroxidase enzyme impaired in inflammatory diseases, and directly counteracting oxidant species. In this view, Maiocchi et al. exploited the TEMPO nucleus to attach different polyamines in order to increase its bioavailability and antioxidant potency [114]. Notably, Put-TEMPO hybrid **70** (Figure 20) achieved one of the highest cellular uptakes, which allowed it to retain efficient inhibition of cellular myeloperoxidase activities such as chlorination, H_2_O_2_ consumption, HOCl production, protein nitration and NO oxidation [114]. Minoxidil belongs to a similar nitrone family and is an antihypertensive agent with antinflammatory properties. Therefore, two series of conjugates with polyamine directly attached to a minoxidil amine functional group or through a urea bridge were developed and evaluated for their antioxidant/antinflammatory properties [115]. From the first series, the Spm conjugate **71** (Figure 20) was derived as a more potent lipid peroxidation inhibitor (94% inhibition at 100 µM) with a generally mild antioxidant profile as well as lipoxygenase inhibition and considerable cytotoxicity in vitro. A similar profile was obtained also for the best urea derivative **72** (Figure 20), with an ameliorated antioxidant profile, except for lipid peroxidation, but in this case, it showed the highest antinflammatory activity in a rat model of acute inflammation (36.5% vs. 22% of **71** at 0.01 mmol/kg measured as inhibition of paw edema). To note, all polyamine-minoxidil conjugates were still less potent than minoxidil alone [115].

## 5. Neuroprotective

Complex pathologies such as cancer and neurodegenerative diseases require suitable and multifaceted treatments to obtain an efficient therapeutic effect. In the latter case, the required brain–blood barrier permeation further complicates the intended strategy. In this field, besides the usual antioxidant profile, polyamine conjugation has been mainly exploited to optimize target interactions or deliver bioactive payloads at specific subcellular compartments, although the mechanisms involving polyamine uptake and trafficking at a central level are still controversial.

**Figure 21:**
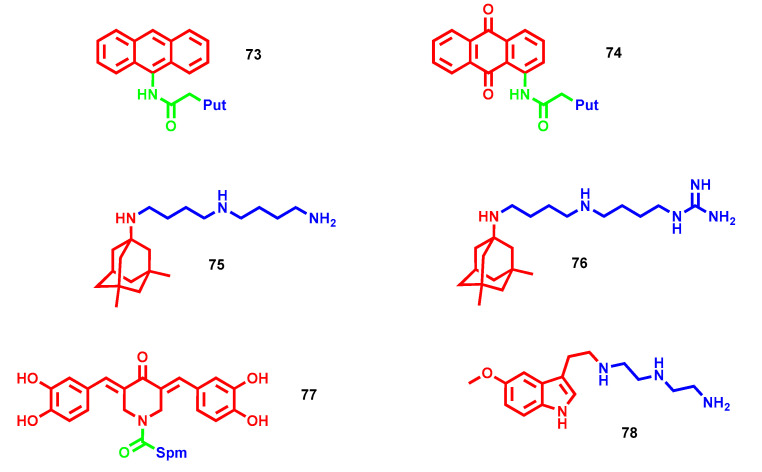
Neuroprotective polyamine conjugates. The bioactive core is highlighted in red, the polyamine chain in blue, and the linker portion in green.

Cholinesterases, such as acetylcholinesterase (AChE) and butyrylcholinesterase (BChE), are enzymes responsible for acetylcholine cleavage, and their inhibition, with the consequent enhancement of cholinergic transmission, represents the therapeutic approach most widely exploited for the treatment of Alzheimer’s disease, albeit only palliative. Inspired by the structure of tacrine, the first cholinesterase inhibitor placed into clinical use and then withdrawn due to hepatotoxicity, several polycyclic polyamine conjugates were developed and screened as ChEs inhibitors [116]. In particular, three aromatic polycyclic building blocks, i.e., naphthalene, anthracene and anthraquinone, were selected and bound to different polyamine moieties. Most of the synthesized molecules are active against ChEs: in particular, anthraquinone–polyamine conjugates are more active on AChE, anthracene–polyamine conjugates are more selective towards BChE, and naphthalene–polyamine conjugates display generally low activity. Compound **73** (Figure 21), bearing a four-methylene linker, is the best one in terms of BChE inhibition, displaying an IC_50_ of 16 nM for this enzyme while being almost inactive for AChE, with a selectivity index (BChE/AChE) higher than 3125. On the other hand, the anthraquinone-Put analogue **74** (Figure 21) turned out to be as the most potent and selective AChE inhibitor (IC_50_ = 1.50 µM). Further kinetic investigations on **73** highlighted interactions with both catalytic and peripheric active sites of the BChE enzyme. Interestingly, anthraquinone **74**, a less potent AChE inhibitor than tacrine, showed higher hepatoxicity in comparison to this drug, while no toxic issues were encountered with anthracene **73**, a more potent BChE inhibitor than tacrine.

*N*-Methyl-*D*-aspartate receptors (NMDARs) play a key role in regulating learning and memory functions, as well as neuroplasticity; however, their glutamate overexcitation, as in the case of AD, leads to excessive calcium influx, which is responsible for neuronal death. Memantine is one of the medications currently used for symptomatic relief in patients suffering from AD, and its mechanisms of action lie in the uncompetitive antagonism of NMDARs, thus mitigating excitotoxic conditions following overexcitation. In this respect, polyamines act as allosteric modulators of NMDARs and possess a specific recognition site on their extracellular side. To develop more efficient NMDAR blockers and increase the therapeutic efficacy of memantine, polyamine–memantine hybrids were developed by Kumamoto et al. with different lengths and terminal substitutions and evaluated against NMDAR GluN2A and GluN2B subtypes [117]. All of the reported derivates demonstrated lower inhibitory potency relative to memantine, except for triamine **75** and diaminoguanidine **76** (Figure 21). In particular, **76** was found to be the most potent NMDAR channel blocker (IC_50_ GluN2A = 379 nM and IC_50_ GluN2B = 432.7 nM vs. 1.376 µM and 2.099 µM, respectively, of memantine), representing a potential starting point for the development of new therapeutics able to tackle excitotoxicity [117].

Oxidative stress and ROS overproduction are commonly widespread during neurodegenerative processes and constitute a means to further foster synaptic loss. In this context, mitochondria represent the cellular hub for ROS production during physiological oxidative phosphorylation, and even more during neurodegeneration when their physiological functions are impaired. Accordingly, mitochondria-directed ligands have been envisioned as a proper strategy to direct antioxidant payloads at the site of toxicity by using a positively charged driving force, such as polyamine, for suitable targeting thanks to the negatively charged mitochondrial membrane. In this context, a series of Spm and norspermidine tails were previously conjugated with curcumin congener core, 3,5-dibenzylidenepiperidine-4-one (DBP), and validated as mitochondria-directed antioxidant agents with less cytotoxic effects of starting moiety [118]. The most promising compound carrying Spm was then further decorated to provide anti-amyloid activity through the insertion of catechol moieties that have been extensively proven to reduce β-amyloid (Aβ) aggregation and related toxicity [119]. The resulting compound **77** (Figure 21) maintained the previous mitochondrial import capacity with an antioxidant profile and also antiaggregant ability (53% of residual Aβ_42*m*_ at 10 µM) and neuroprotection against Aβ-induced toxicity. Interestingly, from molecular dynamic simulations, it emerged that while the catechol motif acts as a key recognition fragment in amyloid binding, the large number of interactions established by **77** along with the perpendicular pose of its Spm tail to the amyloid β-sheets are believed to boost the antiaggregating activity of this conjugate [119].

Some polyamines, depending on the architecture, possess chelating abilities. For example, triethylenetetramine, a copper chelator, is approved for the treatment of Wilson disease, a genetic disorder where copper accumulation occurs in tissues. Metal dyshomeostasis constitutes a pathological feature of complex pathologies by catalyzing ROS production and triggering protein misfolding processes. Following a multitarget approach, Li and colleagues recently developed triethylenetetramine–melatonin hybrid **78** (Figure 21) to merge the chelating abilities of polyamine and the antioxidant/antinflammatory properties of melatonin and evaluate their synergistic efficacy in an AD mouse model at 0.5 mg/kg [120]. Firstly, **78** retained both beneficial properties of the starting synthons in vivo by reducing copper, pro-inflammatory cytokines and ROS content. Furthermore, it down-regulated both AD misfolding processes by mitigating τ hyperphosphorylation and Aβ plaques with a concurrent stimulation of non-amyloidogenic pathways. Unfortunately, none of the detected beneficial properties resulted in a neuroprotective effect in terms of neuronal activity rescue, requiring further optimization [120].

## 6. Conclusions

Over the past two decades, the strategy of conjugating polyamine tails with different anticancer and antimicrobial agents, as well as antioxidant and neuroprotective scaffolds, has been widely exploited to enhance their pharmacological profiles. From a structural point of view, the resulting molecules are quite different compared to the starting scaffolds, and it is, therefore, difficult to predict their metabolic pathways and the biological activity that can arise from this modification.

Although the examples presented in this review, which covered the last 10 years of the literature, show that most of the conjugates retain the activities of the corresponding payloads, suggesting a similar mechanism of action, in some conjugates the polyamine reduces the activity of the parent compound, indicating that the polyamine backbone interferes negatively in the signaling pathways of the conjugated molecule. In the most successful cases, such as the podophyllotoxin–Spm conjugate F14152 currently in clinical trials, the Spm moiety provides the conjugate with enhanced anticancer activity compared to the original compound, modulating additional cellular targets and enhancing cellular uptake. The application of the conjugating strategy between polyamine and an anticancer drug has led to the most promising results. Indeed, high polyamine transport activity and upregulation of its biosynthesis are hallmarks of aggressive cancers. Therefore, the development of anticancer–polyamine conjugates provides a greater chance for anticancer drugs to achieve higher concentrations in cancer cells and more selective targeting than in normal cells.

In this respect, the role of cellular and subcellular delivery vectors of polyamines depends on the full elucidation of PTS molecular structure and functionality. However, differently from bacteria, fungi and plants, the molecular identity and properties of the polyamine transporters in mammalians are poorly characterized. So far, transporters belonging to the solute carrier (SLC), ATP-binding cassette (ABC) and P5B-ATPase transport families have been highlighted as candidate polyamine transporters, but only a few of them have been biochemically or structurally validated. For this reason, only indirect methods (Chinese Hamster Ovary (CHO) cells/PA transport deficient CHO-MG cells or DFMO (difluoromethylornithine)/Spd experiments) are used to evaluate PTS involvement. The recent advances in elucidating the structure of the different polyamine transporters imply the potential of structure-based drug design, not only of drug–polyamine conjugates, but also of PTS modulators to control polyamine homeostasis in the cells that are known to be altered in several pathological conditions [121]. Although the carrier (such as PTS) should be expected to favorably recognize biogenic polyamines (Put, Spd and Spm), the best feature of the polyamine chain that leads to an improvement in the biological activity of the conjugates is unpredictable. Indeed, the examples reported in this review showed that polyamine conjugation contributes not only to improving targeted delivery thanks to active transport systems, but also to the interaction with the target of interest and the overall lipophilicity of the conjugate thanks to the peculiar properties provided by the polyamine moiety. Furthermore, several deficiencies in efficacy were encountered, and also herein reported, in in vitro-to-in vivo translation due to tissue-selective transporters, which recalls the need for proper preclinical characterization.

Despite these pros and cons, the reported summary on the polyamine conjugation approach demonstrated that it is still widely exploited in different and multifaceted drug discovery programs. Further investigations into issues such as the polyamine trafficking in diseased conditions or the metabolic pathways of polyamine conjugates could further boost the potential future clinical translation of this class of compounds.
